# Wearable Sensors for eLearning of Manual Tasks: Using Forearm EMG in Hand Hygiene Training

**DOI:** 10.3390/s16081221

**Published:** 2016-08-03

**Authors:** Ekaterina Kutafina, David Laukamp, Ralf Bettermann, Ulrik Schroeder, Stephan M. Jonas

**Affiliations:** 1 Department of Medical Informatics, Uniklinik RWTH Aachen, Pauwelsstrasse 30, 52057 Aachen, Germany; david.laukamp@rwth-aachen.de (D.L.); ralf.bettermann@rwth-aachen.de (R.B.); sjonas@mi.rwth-aachen.de (S.M.J.); 2 Faculty of Applied Mathematics, AGH University of Science and Technology, Mickiewicza 30, 30-059 Cracow, Poland; 3 Computer Supported Learning Group, RWTH Aachen University, Ahornstrasse 55, 52074 Aachen, Germany; schroeder@informatik.rwth-aachen.de

**Keywords:** Myo armband, eLearning, gesture recognition, surface EMG, smart wearable sensors, hand hygiene, hospital-acquired infections, nosocomial infections

## Abstract

In this paper, we propose a novel approach to eLearning that makes use of smart wearable sensors. Traditional eLearning supports the remote and mobile learning of mostly theoretical knowledge. Here we discuss the possibilities of eLearning to support the training of manual skills. We employ forearm armbands with inertial measurement units and surface electromyography sensors to detect and analyse the user’s hand motions and evaluate their performance. Hand hygiene is chosen as the example activity, as it is a highly standardized manual task that is often not properly executed. The World Health Organization guidelines on hand hygiene are taken as a model of the optimal hygiene procedure, due to their algorithmic structure. Gesture recognition procedures based on artificial neural networks and hidden Markov modeling were developed, achieving recognition rates of 98.30% (±1.26%) for individual gestures. Our approach is shown to be promising for further research and application in the mobile eLearning of manual skills.

## 1. Introduction

Despite the fact that the importance of proper hand hygiene has been known for many decades, it remains a highly prioritized topic for researchers. The PubMed library alone includes almost 1000 scientific papers published within the last 10 years containing the expression “hand hygiene” in the title. The reason for such popularity is simple: despite our theoretical knowledge of the topic, there is still a lot to be improved in real-life situations. The lack of proper hand hygiene is a major healthcare problem, even in developed countries [1,2]. The European Centre of Disease Prevention and Control (ECDC) reports that about 4.1 million EU patients may acquire a healthcare-related infection each year [3]. They directly and indirectly cause an estimated 37,000 and 110,000 deaths, respectively. According to the same source, 20% to 30% of hospital-acquired infections (HAI) are probably preventable by practicing better hand hygiene. This means that the potential health impact of improving the hygiene situation is very high both from a clinical and a politico-economical perspective. According to Malliarou et al., the costs of preventable HAIs in the United States are estimated to be between 2.5 and 5 billion US$ annually [4].

The problem of hand hygiene in hospitals is a complex issue with two main aspects: (1) proper training in the cleaning technique and (2) compliance with disinfection procedures [2]. In other words, if the health care professional is properly trained and willing to perform the disinfection procedure often enough and at the right moments, the hand hygiene requirements should be satisfied and a major cause of HAIs should be eliminated. While compliance has a large impact on hospital hygiene, this work will focus on the training of the technique using smart wearable sensors.

Traditionally, hand hygiene is taught according to the World Health Organization (WHO) guidelines early on in professional medical education [5]. The procedure consists of six basic movements or gestures. The training takes place in a classroom setting, sometimes supported by the use of a fluorescent dye to show cleaning success. The training is rarely repeated at later stages of the career. Even in the early stages, the training is potentially not as effective as it should be. In a preliminary study, we found that only 4 out of 14 nursing students in their second year (after training and first practical experience) covered their hands completely with fluorescent dye and only 5 out of 14 students performed all six gestures defined by the WHO [6].

Meanwhile, the recent decade was very successful for the development of eLearning. Multiple platforms were introduced to support education from the school level, through college, and further in professional life (e.g., Khan Academy, TED, Coursera, Udacity, iTunes University, etc.). There is a great variety of eLearning methods and approaches, but their main focus is on the transfer of theoretical knowledge.

In contrast to theoretical skills, manual training is very challenging—primarily because it usually requires evaluation by an expert professional. This issue has been partially solved for machine operation training using augmented reality [7]. Even in this case, however, no evaluation of pure manual tasks or real-life activities has been possible. With the growing number of easily available mobile sensors, more attention has been drawn to the topic of real life evaluation—for example, the quantified self approach used in fitness and well-being [8,9].

Since skill training with sensors is still in the hatching stage, there are only a very limited number of solutions supporting hand hygiene training. Some examples of market-ready eLearning platforms are the Hand Hygiene Australia system (no mobile version available) [10] and Bio-rite “Hand Hygiene Training”, teaching WHO standards (Android and iPhone versions available). Both of these systems are focused on the theoretical knowledge and do not use any sensor-based feedback loop.

Only one marketed solution working with sensor-based feedback for training is available so far: the SureWash system [11] (Dublin, Ireland). It is aimed at the computer-supported training of the hand hygiene routine. The system is based on pattern recognition from video data and requires a relatively high financial investment. Additionally, the solution is stationary and can only be used in certain environments. The use of a camera for a centralized monitoring system also raises the question of the privacy of employee data. Similar techniques based on video recordings have been proposed by other researchers [12,13].

In general, hand hygiene performance can be recognized in several ways [14,15]. Currently, most hospitals employ staff to manually assess the performance and adherence to hygiene protocols, either personally or through cameras or other sensors. Another common approach to indirectly assess hand hygiene is to measure the amount of dispensed disinfection. This is a very inexpensive measurement but does not provide any feedback on the quality of performance.

Several mobile solutions exist that measure compliance with disinfection procedures. They use mobile sensor-based solutions to track the health professional’s behavior regarding hand hygiene and to point out any “hygiene opportunities” [16,17,18,19]. These sensor networks usually rely on RFID or bluetooth beacons attached to important points in the hospital, and to the hospital staff. However, these systems are not aimed at the training of hand disinfection techniques as such, and act more as reminders than as evaluators.

In addition to a sensor network, Shhedi et al. describe a system to equip staff with motion sensors in a wristband to assess the duration and quality of the hand hygiene procedure, but no evaluation has yet been reported [20]. Similarly, Galluzzi et al. used wrist-worn motion sensors to distinguish between the different gestures of the WHO hand hygiene protocol in a clinical setting [21]. The authors could achieve an accuracy of 89.6% using acceleration and rotation data from both hands.

Since wristbands are usually not permitted in hospitals for hygienic and safety reasons, these systems could be employed as learning tools if modified to not only detect and distinguish between the WHO gestures, but also to evaluate the quality of the performance.

In this paper, we propose the design of an inexpensive mobile platform which will use smart wearable sensors to enable the mobile eLearning of the manual technique used for hand hygiene. We use commercially available armbands to collect data and perform automatic gesture recognition. The evaluation is performed using the WHO guidelines on hand hygiene to define the desired manual task and by applying machine learning methods to analyze the performance of the hand disinfection procedure.

## 2. Materials and Methods

To investigate the feasibility of wearable sensors for the remote and mobile learning of clinical hand hygiene, several steps had to be undertaken: (i) a standard for the training routine had to be selected; (ii) a suitable wearable device had to be chosen; (iii) data of the individual parts of the training routine had to be acquired from several users and labeled; and (iv) a data processing pipeline had to be established and evaluated.

### 2.1. The WHO Standard

**Figure 1:**
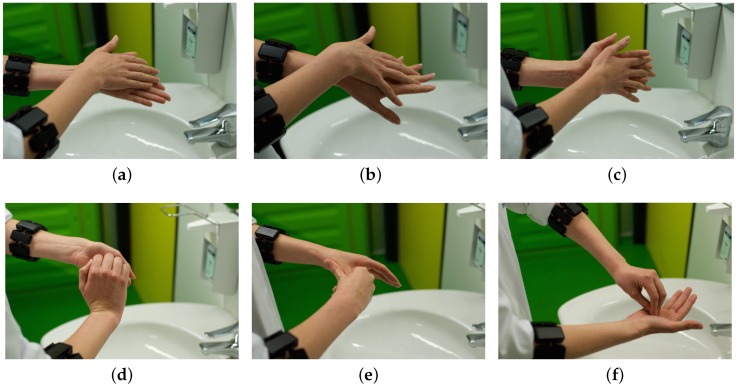
Hand hygiene movements according to WHO: (**a**) G1: cleaning of palms; (**b**) G2L, G2R: cleaning the back of the hands and between fingers; (**c**) G3: cleaning palm and between fingers; (**d**) G4: cleaning back of fingers; (**e**) G5L, G5R: Cleaning thumbs; and (**f**) G6L, G6R: cleaning finger tips and nails. L and R indicate leading left and right hand, respectively.

Typically, hand hygiene in hospitals requires both washing and rubbing with disinfectant, following two almost identical WHO guidelines on hand rub and hand wash [5]. The procedure is very algorithmic: it consists of six basic hand movements (Figure 1). Some of the hand movements are repeated in a symmetrical way with different leading hands, resulting in nine different gestures: (G1) cleaning of palms, (G2L and G2R) cleaning the back of the hands and between fingers, (G3) cleaning palm and between fingers, (G4) cleaning back of fingers, (G5L and G5R) cleaning thumbs, and (G6L and G6R) cleaning finger tips and nails (L and R indicate leading left and right hand, respectively). The recommended time for performance of the full routine is 20–30 s for the hand rub and 40–60 s for the hand wash.

### 2.2. EMG Armband

In this research, gesture control armbands were placed on both forearms to collect arm and hand motion data. Armbands were used as they are not obtrusive in terms of the given task, and they increase the mobility in comparison to stationary devices such as cameras. Here, the commercially available Myo armbands (Thalmic Labs Inc., Kitchener, ON, Canada) are used (see Figure 1). These armbands contain an inertial measurement unit (IMU) and surface electromyography (EMG) sensors. The IMU consists of a three-axis accelerometer and gyroscope, supplying rotation, acceleration, and angular velocity data. Each armband additionally has eight stainless steel surface EMG sensors, which were designed to improve the arm and hand gesture recognition by capturing the muscular activity of the forearm. The device comes with several pre-defined gestures recognized by the original software development kit. The sensor data can be communicated via Bluetooth to other electronic devices.

### 2.3. Data Collection

Data was collected from a group of 17 people who were not medical professionals and had no prior training. The data acquisition followed a strict protocol. Before the acquisition, the participants were instructed by an expert, and the WHO gestures and their order during the procedure were demonstrated. Additional reminders were given before each gesture performance. The armbands were marked to be reproducibly placed on the forearm. Each of the nine gestures was recorded individually for 5 s. The full routine was repeated three times for each person. The position of the Myo armbands was slightly modified between the repetitions in order to better mimic variations in real-life recordings. The data was labeled manually.

The purpose of this session was to collect data which would be usable for the purposes of machine learning to prove the usability of the Myo device as a sensor for training manual skills.

### 2.4. Data Analysis

To investigate the usability of the wearable sensors in the training, a signal processing chain was developed: (1) the sensor data is preprocessed, the sampling frequency is equalized, and the features are extracted; (2) classification of the signal is performed; and (3) hidden Markov models are applied as a post-processing step.

#### 2.4.1. Pre-Processing and Feature Extraction

The IMU and EMG data has to be preprocessed, and features for the later classification have to be extracted. The EMG data is pre-filtered for electrical inference by the Myo armband with a proprietary method. Otherwise, no filtering is applied to any of the signals. The data acquired from the EMG and IMU sensors is sampled with different frequencies (Table 1). In order to be used in classification, the data has to be resampled. Since the acceleration data of the IMU is cumulative, a meaningful upsampling through interpolation cannot be carried out. Thus, the EMG data is downsampled. In order to preserve temporal information, the downsampling is not performed on the raw data prior to feature extraction, but on the feature vectors. This way, temporal information such as high frequencies are preserved and can be used in feature extraction.

As features, wavelet coefficients based on the Daubechies wavelet decomposition and raw data features are created. Different wavelet levels have been tested. The feature vectors were created independently on the IMU and EMG data. The EMG features are then downsampled and aligned with the IMU data. Each final feature vector consists of 16 raw signal values from the EMG, the wavelet coefficients for each of the 16 EMG channels, 20 raw signal values from the IMU, and the wavelet coefficients for each of the 20 IMU channels. For example, a feature vector for a wavelet level of 10 has a dimensionality of 16+16×11+20+20×11=432.

#### 2.4.2. Classification

Artificial Neural Networks (ANNs) were used for the classification of individual gestures. Other techniques, such as *k*-Nearest Neighbors or Support Vector Machines were also tested but performed worse [6,22]. ANNs aim to simulate neuronal functions in the brain and consist of connected and weighted neurons or nodes. Each node has an activation function that determines when a signal is forwarded to the connected nodes. Here, the topology of a feed-forward network with a varying number of layers and nodes is used (see the results for details). The network is trained using scaled conjugate gradient back-propagation and cross-entropy as a performance metric [23]. To accommodate the given multiclass problem, a one-vs.-all approach was used. That is, one neural network is trained for each of the possible classes (nine gestures in our case) to detect exactly this one gesture against any of the others [24]. The class for an input sequence is chosen according to the network with the highest response output. In contrast, the one-vs.-one approach trains classifiers for all possible combinations of two gestures and uses a majority vote or similar scheme to decide on the result.

Five-fold cross validation was used between the 17 individuals for evaluation: the training of the neural network was executed on four parts of the data and the performance was measured on the fifth part. The average error rate of all five possible combinations is reported as a performance metric.

#### 2.4.3. Post-Processing

**Figure 2:**
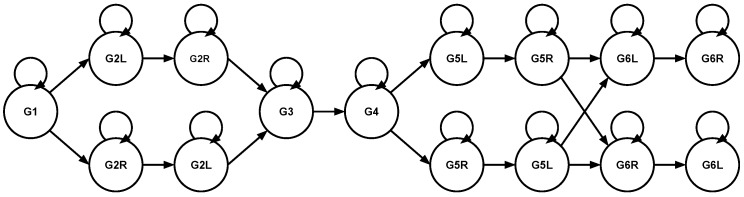
A hidden Markov model (HMM) chain representing the WHO hand washing routine.

Since we have prior knowledge about the underlying procedure, we can use this to improve the results. In our case, switching from one gesture to another several times per second is highly unlikely. We can formalize this knowledge using hidden Markov models (HMMs). HMMs are a machine learning technique successfully used in many similar fields—e.g., in speech recognition [25]. The main idea is to use prior knowledge about the WHO procedure in a state model in which the states correspond to gestures and the transitions between states are associated with a probability. Thereby, frequent transitions between different states (gestures) can be penalized with a low probability, leading to fewer switches between gestures. Additionally, more likely sequences of gestures can be rewarded by assigning them higher probabilities (e.g., between G3 and G4). The predicted sequence is then mapped to a more likely sequence in a global optimization. Here, a fixed model was used that limits the procedure to a predefined sequence with a very low probability of transitioning between any two states (Figure 2).

### 2.5. The Software Employed

An open source software development kit (SDK) was created for the data acquisition with the Myo armband, since the original SDK only supports a single Myo device and is not available for mobile operating systems [26]. The data was processed using Matlab version R2014b (The Mathworks, Natick, MA, USA) and the Hidden Markov Model toolbox for Matlab by Kevin Murphy.

## 3. Results

**Figure 3:**
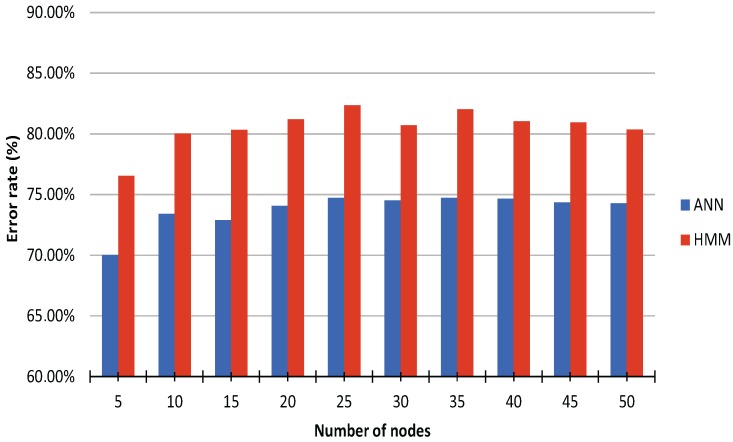
Average recognition rate of artificial neural network (ANN, blue) and HMM (red) using cross validation, wavelet level 3, and a single hidden layer for varying number of nodes in the hidden layer.

**Figure 4:**
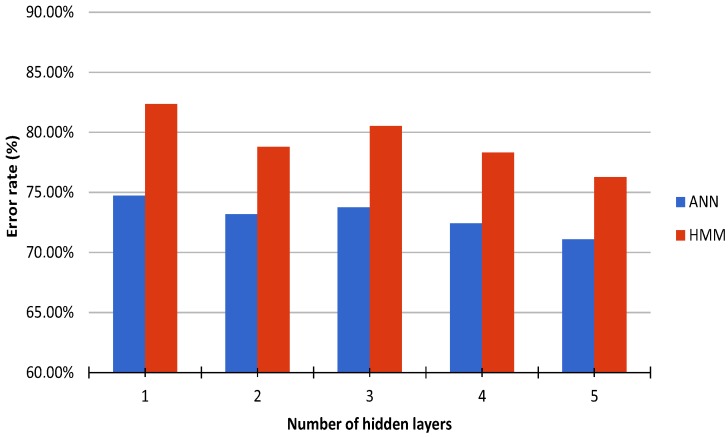
Average recognition rate of ANN (blue) and HMM (red) using cross validation, wavelet level 3, and 25 nodes per layer for varying number of hidden layers.

**Figure 5:**
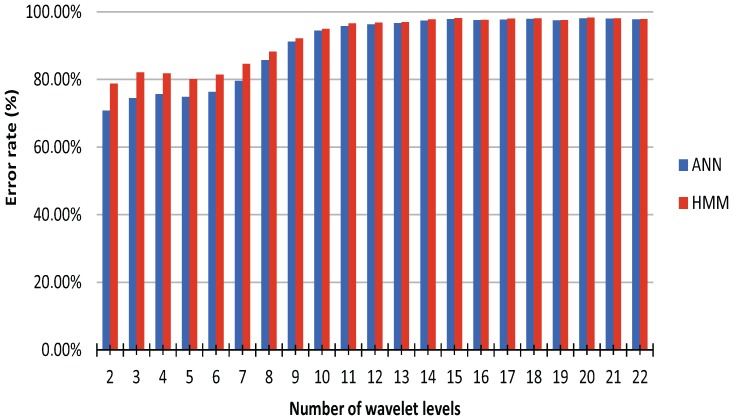
Average recognition rate of ANN (blue) and HMM (red) using cross validation and 25 nodes per layer for varying wavelet levels in the feature extraction.

**Figure 6:**
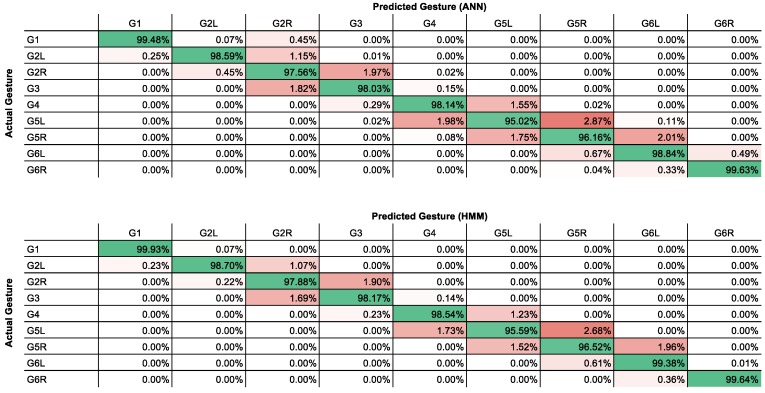
Confusion matrix showing recognition (green) and error (red) rates for best ANN setup before (**Top**) and after (**Bottom**) HMM processing.

The parameters for the ANN classification were optimized empirically in an incremental manner. First, the optimal number of nodes per hidden layer was determined to be 25 (Figure 3). Then, the optimal number of hidden layers was determined to be one (Figure 4). Finally, the optimal number of wavelet levels was determined to be 20 (Figure 5). The error rates for both ANN and HMM are presented to demonstrate the effectiveness of the HMM, especially in cases of lower classification accuracy. The average recognition rate in the best setup for the ANN and HMM corrected sequence was 98.06% (±1.65% standard deviation) and 98.30% (±1.29%), respectively. Most errors are observed between left and right versions of gestures (G5) or subsequent and procedurally similar gestures (G2/G3 and G4/G5) (Figure 6). Errors could be generally reduced by applying a restrictive HMM. The highest recognition rates were observed for gestures G1 and G6.

## 4. Discussion

**Figure 7:**
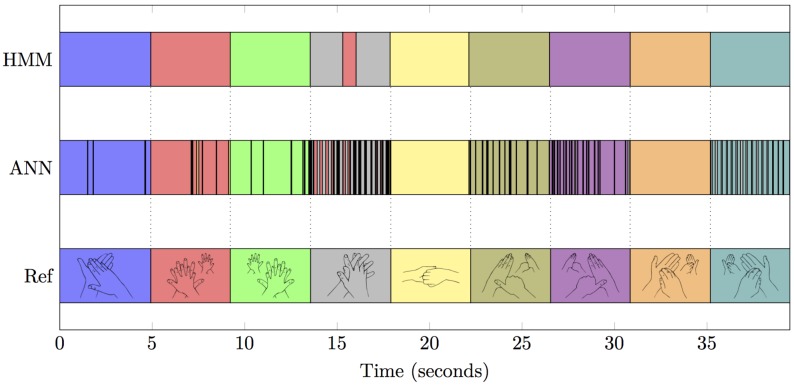
Comparison of the classification results of ANNs with the smoothed version after HMM.

The results presented in the previous section show that the proposed gesture recognition procedure can be successfully implemented in a manual training application using wearable sensors. One of the common problems with low-cost wearable sensors is that the signal-to-noise ratio is typically not very high, which is especially true for surface EMGs. Despite the fact that the chosen pre-processing and feature extraction were very basic, the machine learning methods resulted in an average recognition rate of 98.06% for the chosen setup. The introduction of the hidden Markov modeling (HMM) was able to further smooth the results of the ANN classification, increasing the recognition rate to 98.30% (see Figure 7 for an illustration of the effects of the HMM algorithm). These results outperform other methods based on motion (89.6%, [21]), and are comparable to rates achieved with stationary video-based setups (98.99%, [12]).

Note that the presented parameters were not achieved through a global optimization process, but the resulting error rate is already considered suitable for our purposes. While the chosen setup uses wavelets with a level of 20, even lower values (e.g., level 10) already achieve an error rate below 5% while reducing the computational load significantly. The HMM is able to increase the accuracy greatly, especially when the classification accuracy is below 80%; the good classification results alone indicate that the chosen method might be able to detect gestures of less-structured processes. These results indicate that wearable off-the-shelf sensors can be used for the eLearning of structured and possibly of unstructured tasks, potentially even in a mobile setting with reduced computational power.

We encountered several limitations, which could be overcome in the future. In hidden Markov modeling, we assume that the gestures are performed in a pre-defined order. This assumption could be left out, but a more complex hidden Markov model would then be required. In a real-life teaching situation, several additional problems might occur which should be considered and prevented. As in the case of a modified order of the gesture, more flexibility in the algorithm will be required to overcome individual differences in EMG signal quality—e.g., due to differences in body conductivity from hair or dermal fat. In addition, the position of the Myo armband was determined by an expert, with only little fluctuations between the recordings. A calibration procedure for rotations and translations of the armband should be developed to overcome this limitation.

The current algorithm is implemented in Matlab, which allows a very good control of the subsequent steps of the calculations and the use of the built-in packages for machine learning methods. On the other hand, the speed of execution could be improved, which is necessary for real-time use. This will be done in later stages of the project.

Lastly, note that the signal processing and feature extraction methods used in this work could be further optimized, which might influence the computation time, error rate, and generalization qualities of the system.

## 5. Conclusions

The goal of this manuscript was the design and implementation of a signal processing framework for sensor-based gesture recognition in WHO hygiene routine training. The framework was successfully developed and shown to be suitable for further research. The data from the IMU and surface EMG sensors built into Myo Thalmic armbands was used to develop an effective gesture recognition algorithm. The low resulting error rates indicate that smart wearable sensors could be successfully used in the training of manual skills. This area of eLearning is still in the initial stages of its development and can greatly benefit from the reported results.

Based on the presented data processing, an eLearning software will be developed using a serious game approach containing traditional eLearning materials (such as instructional videos, texts, quizzes), but most importantly focussing on manual training and direct feedback. The biggest challenges of the application will be the scoring algorithm, as it is not a trivial task to draw a parallel between the effectiveness of the actual hand hygiene procedure and a correct gesture. The effectiveness of the eLearning application will be evaluated in two clinical trials. The first trial should investigate the research question of how subtle differences in the routine and gestures can influence the hand washing result and how is this reflected in our EMG readings. Here, users should wash their hands according to protocol and protocol-variations with fluorescent dye. The coverage of the hands should then be measured and correlated with the performance of different gestures and recognized adherence to the predefined gesture routine. The second trial should be a long-term randomized clinical trial to answer the research question of whether the proposed system can lead to improved hand hygiene in comparison to traditional training and reminders, as measured via proxies (e.g., disinfectant usage, HIA incidence rate). One could also investigate whether our system might be preferable, in terms of motivation or privacy, to personal training or regular reminders.

Finally, integration into clinical education or even regular check-ups for qualified nurses, paramedics, and doctors using the eLearning game can be proposed (e.g., as monthly leaderboard challenges). In this case, a serious game might yield a higher acceptance than traditional courses or regular training settings in a classroom environment [27]. In fact, during the preliminary data collection from nursing students, 85% of the students would use such a system as a part of their education and 25% even in a mobile setup or at home. The most desired output would be to foster a long-term habit of hand hygiene, thereby also improving the issue of hygiene compliance in clinical routine.

## Figures and Tables

**Table 1:** Overview of the sensors and data types. EMG: electromyography; IMU: inertial measurement unit.

	EMG	IMU
Sampling rate	200	50
#Values	8	10
Data type	int8	float

## Abbreviations

The following abbreviations are used in this manuscript:

ANN: Artificial Neural Network
ECDC: European Center of Disease Prevention and Control
EMG: ElectroMyoGraph
HMM: Hidden Markov Model
IMU: Inertial Measurements Unit
SDK: Software Development Kit
WHO: World Health Organization
