# Correction: Kutafina, E.; Laukamp, D.; Bettermann, R.; Schroeder, U.; Jonas, S.M. Wearable Sensors for eLearning of Manual Tasks: Using Forearm EMG in Hand Hygiene Training. *Sensors* 2016, *16*, 1221

**DOI:** 10.3390/s19214792

**Published:** 2019-11-04

**Authors:** Ekaterina Kutafina, David Laukamp, Ralf Bettermann, Ulrik Schroeder, Stephan M. Jonas

**Affiliations:** 1 Department of Medical Informatics, Uniklinik RWTH Aachen, Pauwelsstrasse 30, 52057 Aachen, Germany; david.laukamp@rwth-aachen.de (D.L.); ralf.bettermann@rwth-aachen.de (R.B.);; 2 Faculty of Applied Mathematics, AGH University of Science and Technology, Mickiewicza 30, 30-059 Cracow, Poland; 3 Computer Supported Learning Group, RWTH Aachen University, Ahornstrasse 55, 52074 Aachen, Germany

The authors wish to make the following erratum to this paper [1]:
